# Endogenous Modulators of NMDA Receptor Control Dendritic Field Expansion of Cortical Neurons

**DOI:** 10.1007/s12035-022-03147-0

**Published:** 2022-12-03

**Authors:** Pascal Jorratt, Jan Ricny, Christian Leibold, Saak V. Ovsepian

**Affiliations:** 1 National Institute of Mental Health, Klecany, Czech Republic; 2 Third Faculty of Medicine, Charles University, Prague, Czech Republic; 3 Faculty of Biology and Bernstein Center Freiburg, University of Freiburg, Freiburg, Germany; 4 Faculty of Science and Engineering, University of Greenwich London, Chatham Maritime, Kent, ME4 4TB UK

**Keywords:** Dendritic morphology, psd95, Neurosteroids, Polyamines, Glutamate release, Cortical thinning, Intrinsic excitability, Neuroplasticity

## Abstract

Impairments of *N*-methyl-*D*-aspartate receptor (NMDAR) activity have been implicated in several neuropsychiatric disorders, with pharmacological inhibition of NMDAR-mediated currents and associated neurobehavioral changes considered as a model of schizophrenia. We analyzed the effects of brief and long-term exposure of rat cortical cultures to the most prevalent endogenous modulators of NMDAR (kynurenic acid, pregnenolone sulfate, spermidine, and zinc) on neuronal viability, stimulation-induced release of glutamate, and dendritic morphology with synaptic density. Both, glutamate release and neuronal viability studies revealed no difference between the test and control groups. No differences were also observed in the number of dendritic branching and length, or density of synaptic connections and neuronal soma size. Comparison of the extent of dendritic projections and branching patterns, however, revealed enhanced distal arborization with the expansion of the dendritic area under prolonged treatment of cultures with physiological concentrations of NMDAR modulators, with differences reaching significance in spermidine and pregnenolone sulfate tests. Measurements of the density of glutamatergic synapses showed consistency across all neuronal groups, except those treated with pregnenolone sulfate, which showed a reduction of PSD-95–positive elements. Overall, our data suggest that constitutive glutamatergic activity mediated by NMDAR controls the dendritic field expansion and can influence the integrative properties of cortical neurons.

## Introduction

Excitatory neurotransmission in the central nervous system (CNS) is mediated mainly by glutamate, which activates ionotropic and metabotropic glutamatergic receptors. The ionotropic receptors comprise multiple subtypes of α-amino-3-hydroxy-5-methyl-4-isoxazole propionic acid (AMPA), kainite (KA), and *N*-methyl-*D*-aspartate (NMDA) receptors, which are widely expressed along the entire axis of CNS [1]. Unlike AMPA and KA receptors activated by glutamate alone and passing almost exclusively Na^+^ ions, activation of NMDA receptors (NMDAR), in addition to glutamate, requires membrane depolarization with the removal of Mg^2+^ block of the NMDAR-channel and coactivation by glycine/d-serine, to mediate composite Na^+^/Ca^2+^ currents [2, 3]. These unique features render the NMDAR-mediated transmission responsive to concurrent activation of presynaptic (glutamate release) and postsynaptic (membrane depolarization) elements of glutamatergic synapses (i.e., coincident detection), with the rise in intracellular Ca^2+^ triggering mechanisms of structural and functional synaptic plasticity [4].

The alleged central role of NMDAR in synaptic plasticity renders it of prime interest for studies of the neurobiology of learning and memory, with impairments linked to the cognitive decline and loss of memory in Alzheimer’s disease and related dementias, as well as to several neuropsychiatric conditions [5, 6]. There is ample evidence suggesting NMDAR deficiency in schizophrenia, with dissociative anaesthetics antagonizing NMDAR activity used for inducing (and exploring) schizophrenia-like symptoms in humans and animal models [7–9]. Evidence from human brain autopsies shows significant morphological alteration of pyramidal neurons in patients with chronic schizophrenia, including reduced complexity of dendritic trees in the prefrontal cortex and other brain structures, which contribute to cortical thinning and brain atrophy [10–12]. Histopathological analysis also suggests a reduction in the density of synaptic contacts in cortical tissue, with results of morphometric studies implying loss of dendritic spines and glutamatergic synaptic connections [13, 14].

Although much research has been carried out on the effects of ketamine, phencyclidine, MK801, and other exogenous antagonists of NMDAR on neuronal excitability, synaptic functions, and plasticity, little is known about the role of endogenous modulators [15]. Impairments of NMDAR activity in schizophrenia and several other neuropsychiatric diseases [15–20] urge in-depth analysis of underlying mechanisms and functional implications. We studied the effects of brief and prolonged exposure of rat cortical neurons to physiological concentrations of endogenous NMDAR modulators—kynurenate (KYNA), pregnenolone sulfate (PS), spermidine (SPD), and zinc (ZINC). We measured the glutamate release, neuronal viability, morphology and branching of dendrites, and synaptic density and report important changes in the expansion of the dendritic tree with potential effects on neuronal properties and functions. Collectively, our data suggest a new neurobiological mechanism connecting the impairments of NMDAR-mediated glutamatergic activity with schizophrenia and other neuropsychiatric disorders associated with a deficiency of NMDAR functions.

## Materials and Methods

### Primary Cortical Cultures

**Fig. 1:**
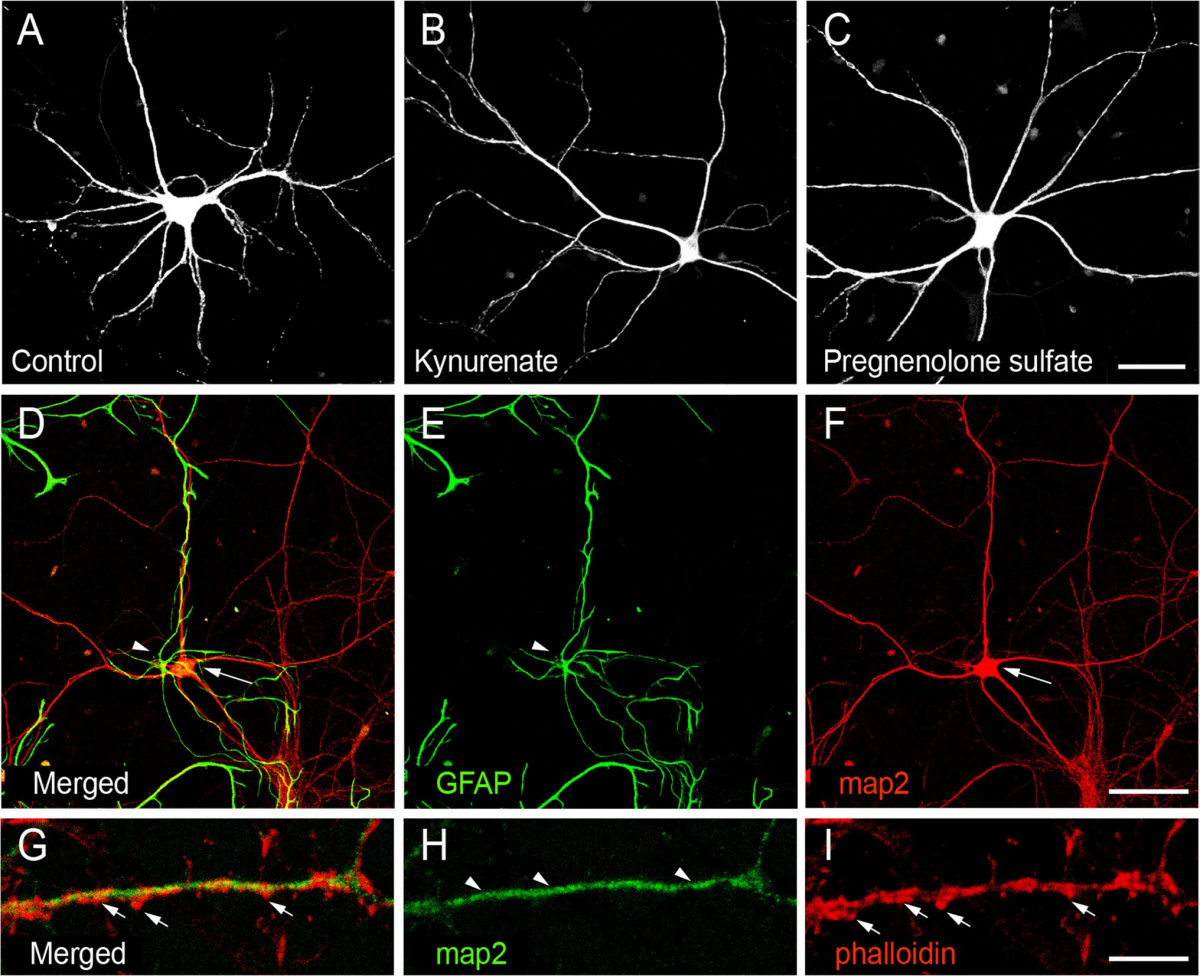
Optimization of protocols for morphometric analysis of neurons in cortical cultures. **A**–**C** Representative confocal micrographs (*z*-projections) of cortical neurons (from left to right: control, kynurenic acid, and pregnenolone sulfate treated) stained for map2 protein. Scale bar: 40 µm. **D**–**F** Micrographs of GFAP and map2 double-stained cortical culture (div19). Scale bar 150 µm. Arrow points to the soma of a neuron; the arrowhead points to a GFAP-positive astrocyte. **G**–**I** A fragment of a dendritic branch of cortical neuron stained for map2, bearing multiple phalloidin-positive dendritic spines. Scale bar 7 µm

All experimental procedures have been approved by the NIMH Committee for Animal Research Ethics in line with the Animal Protection Code of the Czech Republic and the directive of the European Community Council (2010/63/EU). Primary cortical cultures were prepared from embryonic day 18 Wistar rats, which were extracted from timed pregnant dams. Embryos (d18) were decapitated using a guillotine with brains gently removed, followed by isolation of cortices and incubation in ice-cold Hank’s balanced salt solution (HBSS) (Thermo Fisher Scientific, 14175095). After 3 washes in HBSS, tissue was dissociated mechanically using a 0.9-mm needle (B. Braun, 4657519) followed by a 0.45-mm needle (B. Braun, 4657683). The tissue suspension was subsequently passed through a 100-μm pore nylon filter (VWR, 732–2759) and re-suspended in a seeding medium, which contains DMEM (Biowest, L0104-500) supplemented with 10% FBS (Biowest, S1810-500) and 1% Penicillin–Streptomycin (Thermo Fisher Scientific, 15070063). For immunohistochemical experiments, dissociated neurons were plated at 20,000 cells/cm^2^ density on 13-mm coverslips (Marienfeld-Superior, 0117530) coated with poly-L-lysine in 24-well plates. For glutamate release and viability assays, neurons were plated at a higher, 125,000 cells/cm^2^ density on coated 24- and 96-well plates. All cultures were maintained at 37° C and 5% CO_2_. During the next day, the seeding medium was replaced with a growth medium, which contained a Neurobasal medium (Thermo Fisher Scientific, 21103049) with 1% Penicillin–Streptomycin, 2-mM L-Glutamine (Thermo Fisher Scientific, 25030149), and 2% B27 serum-free supplement (Thermo Fisher Scientific, A3582801). Every 4–5 days, half of the growth medium was removed and replaced with a fresh medium. No inhibitors of cell proliferation have been added to the growth medium; therefore, the material we analyzed contained a mixture of neurons and glial cells, as verified by immunofluorescence staining for neuronal and glial markers (Fig. 1A-F).

### Treatments of Cultures with Endogenous NMDAR Modulators

At days in vitro (div) 14, cultures were treated for 1 or 5 days (brief or prolonged, respectively) with kynurenic acid (KYNA, 150 nM), pregnenolone sulfate (PS, 50 μM), spermidine (SPD, 50 μM), or zinc (ZINC 10 μM) (all obtained from Sigma-Aldrich). We selected these concentrations to mimic their physiological levels reported in the cerebrospinal fluid [15]. As all used compounds at specified concentrations are water-soluble, we used bi-distilled water as a solvent and vehicle for control experiments. After treatments, cortical cultures were tested for cell viability and stimulation-induced (50 mM K^+^) glutamate release assays or were fixed for immunofluorescence staining and morphometry.

### Neuron Viability Assay

Neuron viability was assessed using 3-(4,5-dimethylthiazol-2-yl)-5-(3-carboxymethoxyphenyl)-2-(4-sulfophenyl)-2H-tetrazolium (MTS) colourimetric assay which detects the conversion of the tetrazolium into a formazan product in living cells. After treatment with endogenous NMDAR modulators (12 h, 1 and 5 days), MTS was added to the cultures (10 µL of MTS to 100 µL of medium) at div19 and incubated for 2 h at 37 °C and 5% CO_2_. Subsequently, the optical density (OD) was measured at 490 nm in a plate reader (Tecan Infinite M200 Pro). The percentage of cell viability was calculated using a formula: (*OD*_sample_/*OD*_control_) × 100. 

### Glutamate Release Measurements from Primary Cultures

For quantifying glutamate concentration, we employed a method with amino acid derivatization by dansyl chloride and fluorescence detection as described in Kristofikova et al. [21] with some modifications. After 1- or 5-day exposure of primary cortical cultures to endogenous modulators of NMDAR, the growth medium was removed, and cells were stimulated for 10 min with 50 mM K^+^ in Krebs–Ringer (KR) solution (75 mM NaCl, 50 mM KCl, 2.5 mM CaCl_2_, 1 mM MgSO_4_, 1 mM NaH_2_PO_4_, 10 mM glucose, 20 mM HEPES pH 7.4, 0.3 mM DL-TBOA). Culture supernatant was afterwards collected and used for glutamate measurement, while cells were dissolved in 0.2 N NaOH for protein measurements as described elsewhere [22], but modified for microplates, with BSA used as standard. For quantifying glutamate concentration, 0.1 ml of supernatant was mixed with 0.1 ml of 200 mM NaHCO_3_/10 mM EDTA and 0.4 ml of dansyl chloride (1.25 mg/ml dissolved in acetone) and reacted in the dark for 1 h/60 °C. Subsequently, samples were lyophilized (Hanil module 4080C centrifuge vacuum concentrator) and reconstituted in 0.2 ml H_2_O. After centrifugation (10 min/15,000 × g/4 °C, Hettich Universal 32R), samples (20 µl) were injected into the HPLC system (Thermo Scientific Dionex Ultimate 3000 with quarternary pump, fluorescence detector, and autosampler), resolved on a C18 column (Separon SGX C18, 3 × 150 mm), with derivatized glutamate monitored using fluorescence detection (330 nm excitation and 550 nm emission). HPLC conditions were optimized for measurements of glutamate. Chromatography was performed isocratically at 0.4 ml/min for 15 min in the “separation” mobile phase (50 mM K-PO4 pH 7.2/8% acetonitrile/14% methanol) followed by the “chase” mobile phase (50 mM K-PO4 pH 7.2/70% methanol) for 5 min and re-equilibration, with “separation” mobile phase for 10 min. Glutamate levels were calculated using calibration method (1.25, 2.5, 5, and 10 μM; i.e., 25, 50, 100, and 200 pmol/injection) and presented after normalization for the protein lysate content, using regression analysis.

### Immunocytochemistry

The cortical cultures were washed with phosphate buffer solution (PBS) for 5 min and fixed with paraformaldehyde (4% in PBS and 2% sucrose) for 15 min, followed by thrice washing with PBS for 5 min each. Background immunoreactivity was blocked and cells permeabilized with 10% fetal bovine serum (FBS) in Triton X-100 0.1% for 1 h. This step was followed by overnight incubation of cultures with chicken anti-microtubule associated protein 2 (map2) (1:10000; Abcam, ab5392), mouse anti–PSD-95 (1:500; Abcam, ab192757), anti–glial fibrillary acidic protein (1:500; GFAP, rabbit polyclonal, 1:5000; Dako, catalogue # Z033429), or guinea pig anti–synapsin I/II (1:1000; Synaptic Systems, 106004). Dendritic spines were stained using Alexa Fluor 405 Phalloidin, which is known to label F-actin enriched in spines (Thermo Fisher, catalogue #A30104) (Fig. 1G-I). After 3 washes, fixed samples were incubated with the secondary donkey-anti-chicken, donkey-anti-mouse, and donkey-anti-guinea pig antibodies conjugated to Alexa Fluor 488, 594, and 647 (1:500; Jackson ImmunoResearch, 703–545-155, 715–605-150, and 706–585-148, respectively) for 1 h followed by rinsing with PBS for 5 min and mounting with FluoroShield (Sigma-Aldrich, F6182), for viewing and confocal imaging. 

### Image Acquisition and Analysis

All experiments were performed in duplicates from three biological replicates. Confocal images were acquired with Leica DMi8 (Leica Microsystems) laser scanning microscope controlled by LAS X software (Leica Microsystems). For illustration purposes, representative neurons were reconstructed manually using GNU Image Manipulation Program (GIMP 2.10, www.gimp.org). For synapse density counting and visualization of spines, images were taken using a 63 × oil objective (NA = 1.4) with Z-stacks at 0.3 μm step size and 2.6 × digital zooms. For dendritic tree morphology studies, images were taken using a 40 × oil objective (NA = 1.3) with Z-stacks at 0.8 μm step size. Binary images of neurons were analyzed using Synapse Counter [23] and SNT v3.2.11 plugin [24] for Fiji. All images had been analyzed by an experimenter blinded to treatment conditions. Binary images were automatically thresholded, and co-localizing puncta were analyzed using Synapse Counter [23]. Neurons were reconstructed using SNT v3.2.11 plugin [24] for Fiji, and 2D Sholl analysis was performed with a 10 µm radius step size from the center of the soma (single point) using the same plugin. All images had been analyzed by an experimenter blinded to treatment conditions.

### Computational Model

The used models of cortical neurons comprised one somatic (surface: *S* = 800 µm^2^) and two passive dendritic (surface: *S* = 10,000 µm^2^ each) compartments with a specific constant leak conductance of *g* = 0.33 mS/cm^2^. The specific capacitance was adjusted to *c* = 1 µF/cm^2^. Dendritic compartments were connected by a length resistance of 3 MΩ (corresponding to the compartments forming cylinders of, e.g., 30 µm length, 6 µm diameter, and 100 Ω/cm specific resistance). The voltage *V*_i_(*t*) of the i-th compartment evolved according to the differential equation

$$dV_{i/dt}=\left[{G}_{i}\left(V_{rest}-{V}_{i}\left(t\right)\right)+1/\left(3\mathrm{M\Omega }\right){\sum }_{j=1}3{M}_{ij}{V}_{j}\left(t\right)+{I}_{\mathrm{i}}\right]/{C}_{\mathrm{i}}$$

with capacitances *C*_i_ = *S*_i_ *c*, transmembrane conductances *G*_i_ = *S*_i_ *g*, connectivity matrix *M*_ij_ (0 or 1) between i-th and j-th compartment (according to the morphological schemas in Fig. 5B), and current clamp input *I* (only applied to a somatic compartment). Spiking was induced by an integrate and fire mechanism for which an action potential was elicited when the somatic membrane voltage crossed the threshold of 10 mV (relative to resting potential) from below. After each such threshold crossing, the neuron model was set into an absolute refractory state over 2.5 ms, during which no further input was integrated, and an action potential template was pasted into the somatic voltage traces for visualization. After the refractory period, the voltage was reset to resting potential. Simulation parameters were adjusted to achieve typical values for input resistances, thresholds, and maximum firing rates of cortical neurons as reported elsewhere [25]. The python file by which simulated data and panels from Fig. 5 were generated can be found in the github repository (cleibold/passivedendrites).

### Data Analysis and Statistics

Data in all bar graphs are represented as mean ± SD. One-way ANOVA followed by Dunnett’s post hoc or two-way ANOVA followed by Tukey’s post hoc test was used to compare the different treatments to the control. Shapiro–Wilk test was performed to evaluate the data distribution, with non-normal distributed data transformed to Log (X + 1) to satisfy the requirements of the ANOVA model. The significance threshold was set at *p* < 0.05, with values below considered statistically significant. All statistical tests were performed using R software (version 4.0.5) and RStudio (version 1.4.1717).

## Results

**Fig. 2:**
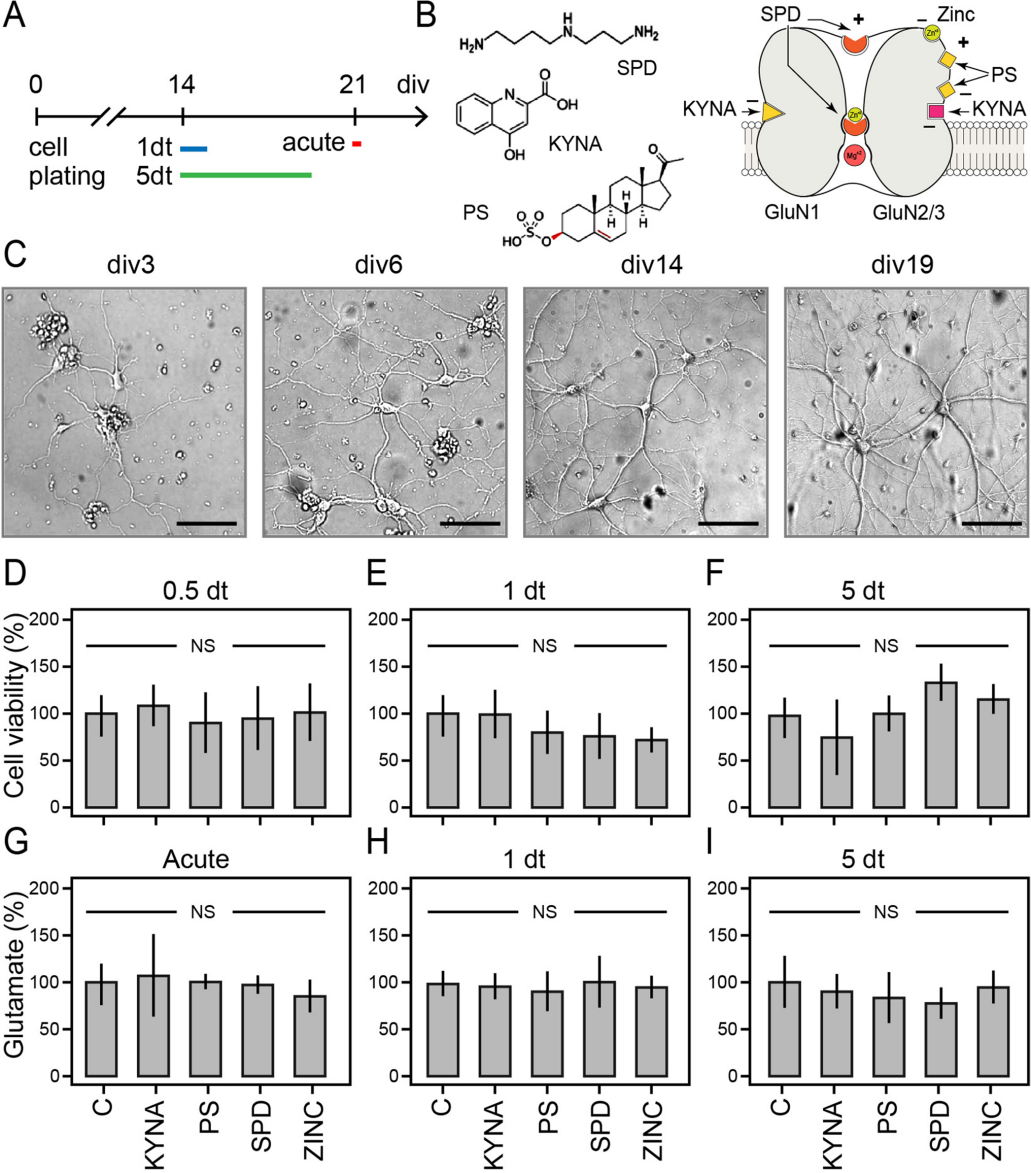
Analysis of cell viability and glutamate release of cortical cultured neurons treated with endogenous NMDAR modulators. **A** Timeline of the treatments in cortical cultures. Abbreviations: dt, days of treatment; div, days in vitro. **B** Chemical structure of the endogenous modulators of NMDA receptor and schematized sites of binding to NMDAR. **C** Low power images of cortical cultures at div 3, 6, 14, and 19. Scale bar: 80 µm. **D**–**F** Results of MTS assay for cell viability performed after 12-h (0.5 dt) and 1 and 5 dt. *N* = 6–7 technical repeats from two biological replicas. **G**–**I** Summary plots of KCl-evoked glutamate release of cortical culture. *N* = 5–6 technical repeats from two biological replicas. The control level is taken as 100%. Data are normalized by the protein lysate content and represented as means ± S.D. One-way ANOVA. NS, non-significant

To investigate the effect of endogenous NMDAR modulators on neuronal viability, morphology, and functions, we carried out viability tests, functional assays of glutamate release, and morphometric analysis in cortical cultures after their brief or prolonged exposure to KYNA, PS, SPD, and ZINC and compared the readouts with age-matched untreated cortical cultures. Figure 2A-C illustrates the experimental design with a chemical structure of used endogenous modulators and representative examples of primary cortical cultures at different developmental stage.

## Endogenous NMDAR Modulators Do Not Alter Cell Viability and Glutamate Release

Given the key role of NMDAR in a wide range of signalling processes governing neuronal development, activity, and synaptic plasticity, first, we carried out MTS viability tests to determine if the exposure of neurons to endogenous NMDAR modulators causes any toxicity or alters the neuronal survival. After 12-h and 1- or 5-day treatment of cultures with KYNA, PS, SPD, or ZINC, MTS assay revealed no change in the cell viability (one-way ANOVA; 12 h *F*_(1,4)_ = 0.36, *p* = 0.834; 1 day *F*_(1,4)_ = 1.56, *p* = 0.211; 5 days *F*_(1,4)_ = 1.11, *p* = 0.370) (Fig. 2D-F). This result demonstrates that at physiological concentrations, KYNA, PS, SPD, and ZINC do not affect the viability of neurons in primary cortical cultures. Next, we tested if exposure of cortical neurons to endogenous NMDAR modulators affects the release of glutamate (Fig. 2G-I). The differences between basal glutamate levels in cortical cultures untreated and treated with KYNA, PS, SPD, and ZINC were within the variation range; therefore, we turned to measurements of the evoked release of glutamate in the presence of an inhibitor of glutamate uptake DL-TBOA [26]. In the first set of experiments, the glutamate release was measured after cultures were exposed to a high K^+^ Krebs–Ringer (KR) solution for 10 min together with NMDAR modulators. A comparison of the released glutamate with that without K(+ superscript) stimulation revealed no differences (Fig. 2A, G). Next, we measured evoked glutamate release by high K^+^ Krebs–Ringer solution in cultures after their 1 or 5 days of exposure to endogenous NMDAR modulators. The results of these studies were similar to those obtained with co-treatment of culture with high-K^+^ and NMDAR modulators. The amount of evoked glutamate release, after normalizing for the protein yielded by cultures, was consistent across all groups (one-way ANOVA; acute *F*_(1,4)_ = 0.39, *p* = 0.818; 1 day *F*_(1,4)_ = 0.56, *p* = 0.694; 5 days *F*_(1,4)_ = 0.24, *p* = 0.915) (Fig. 2G-I).

## Endogenous NMDAR Modulators Promote the Dendritic Expansion

**Fig. 3:**
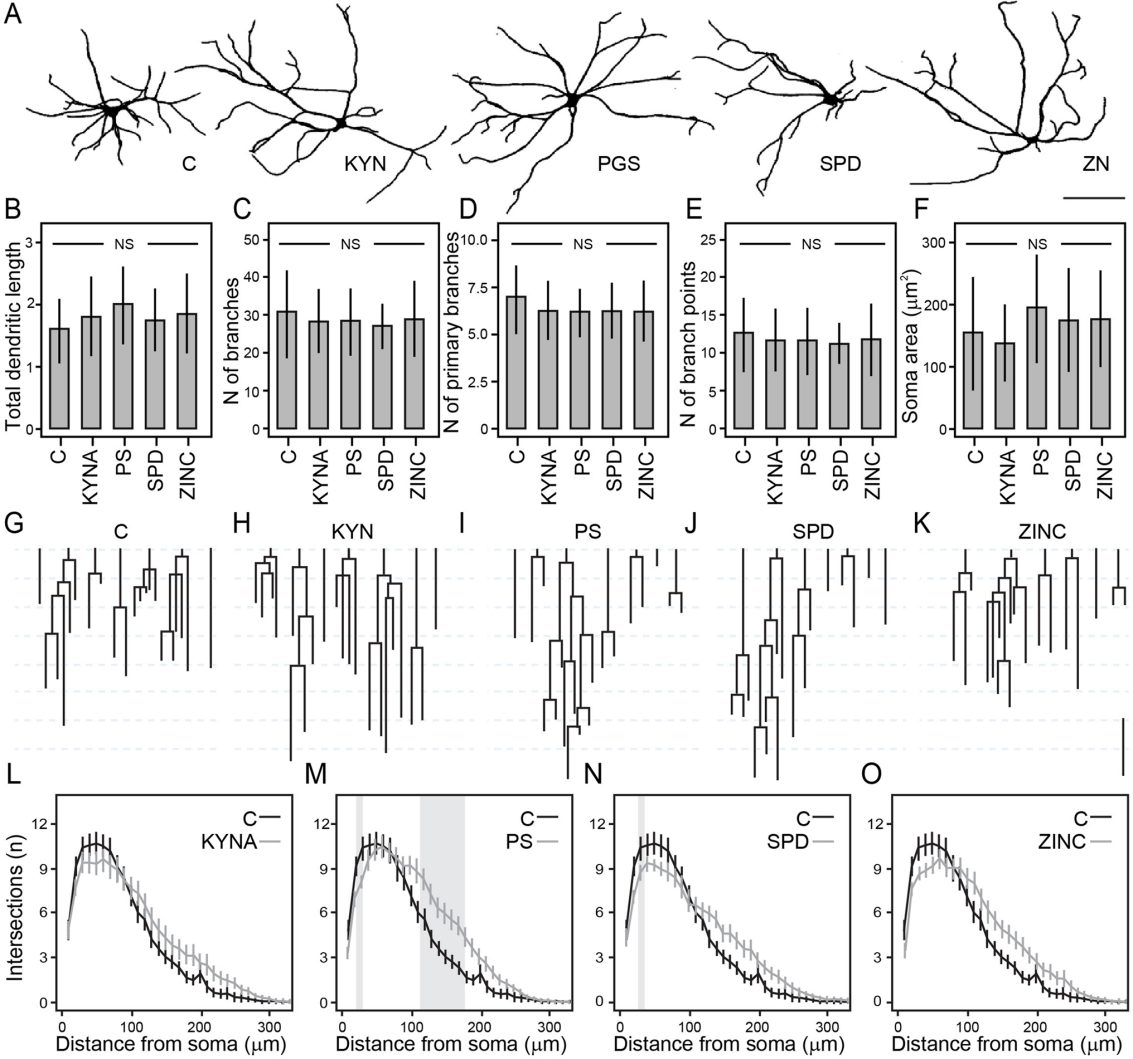
Analysis of dendritic branching and arbor complexity in cortical neurons treated for 5 days with different NMDAR modulators. **A** Representative examples of reconstructed neurons. **B**–**F** NMDAR modulators did not produce a difference in the **B** total dendritic length, **C** number of branches, **D** number of primary branches, **E** number of branch points, and **F** soma area. Classical dendrogram representation of the dendritic branching of representative neurons schematized as 2D binary trees (**G**–**K**) and results of Sholl analysis (**L**–**O**) showing the number of intersections along with the distance from the soma for different treatments. The difference within the gray region is statistically significant (*p* < 0.05). Data are represented as means ± S.D. *n* = 23–30 randomly chosen neurons per treatment from three biological replicas. One-way ANOVA followed by Dunnett’s post hoc. Scale bar = 100 μm

The uniformity of depolarization-induced release of glutamate (normalized against total protein) across all treatment conditions suggests that the regulated (action potential–dependent) synaptic transmission has not been affected by NMDAR modulators. Given the importance of the NMDAR in shaping synaptic functions and plasticity, we investigated if brief or prolonged exposure of cortical cultures to KYNA, PS, SPD, and ZINC can cause any measurable structural alterations. Div 14 cultures were exposed to and treated with NMDAR modulators for 1 or 5 days and fixed for morphological changes, including measurements of dendritic branching and arborization index, total length, and neuronal soma size (Fig. 3A-F). Under all pharmacological treatments, the total dendritic length (Fig. 3B), the total number of dendritic branches (Fig. 3C), the number of primary branches (Fig. 3D), the number of branching points (Fig. 3E), and neuronal soma size (Fig. 3F) remained unchanged (one-way ANOVA; total dendritic length *F*_(1,4)_ = 1.13, *p* = 0.348; number of branches *F*_(1,4)_ = 0.57, *p* = 0.682; the number of primary branches *F*_(1,4)_ = 1.28, *p* = 0.281; the number of branch points *F*_(1,4)_ = 0.39, *p* = 0.816; soma area *F*_(1,4)_ = 3.06, *p* = 0.019) (no difference between groups and control in Dunnett’s post hoc test). However, using Sholl analysis, we observed differences between the numbers of neurite crossings of the concentric circles in 5-day–treated cortical neurons, with effects depending on the distance from the soma (Fig. 3G-K). In all drug-treated cortical cultures, the number of crossings proximal to the soma (< 100 µm) showed a trend for reduction as compared to controls, whereas the number of crossings of distal neurites (> 100 µm) showed a strong trend for an increase in branching as compared to controls (Fig. 3L-O). The differences in mean values in these tests reached statistical significance in cortical cultures exposed to PS and SPD over 5 d. While both PS and SPD decreased significantly the number of intersections at 20 µm (one-way ANOVA; *F*_(1,4)_ = 2.51, *p* = 0.045), only PS increased significantly the number intersections at 130, 150, 170, and 180 μm (one-way ANOVA; 130 μm *F*_(1,4)_ = 2.55, *p* = 0.042; 150 μm *F*_(1,4)_ = 2.74, *p* = 0.031; 170 μm *F*_(1,4)_ = 2.80, *p* = 0.029, 180 μm *F*_(1,4)_ = 2.60, *p* = 0.040). From these experiments, it emerges that at physiological concentrations, endogenous modulators of NMDAR facilitate dendritic arborization in remote compartments and promote the expansion of the dendritic projections.

## Endogenous NMDAR Modulators Do Not Alter the Synaptic Density of Cortical Neurons

**Fig. 4:**
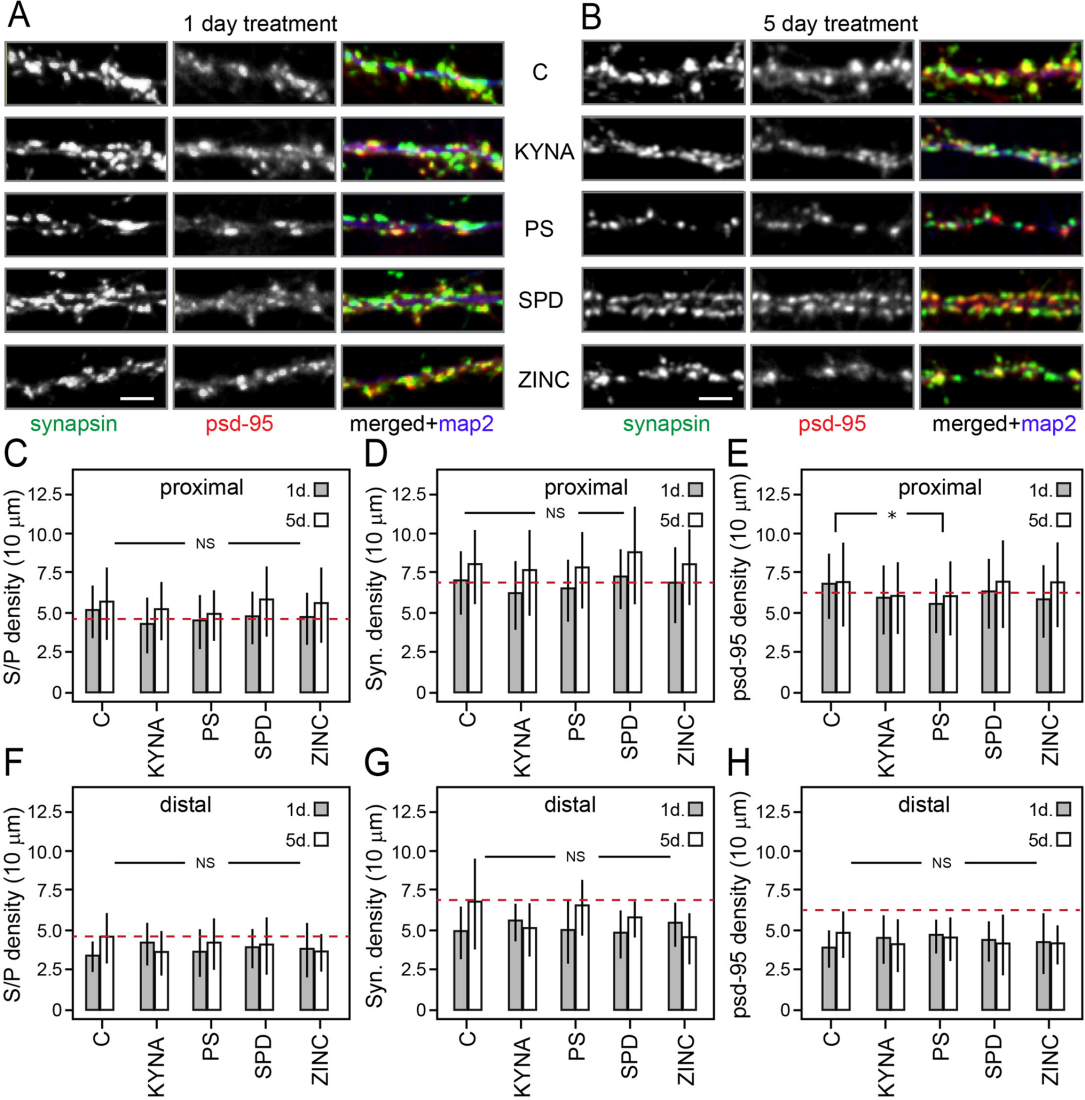
Analysis of synaptic density and presynaptic terminals in cortical cultures treated for 1 or 5 days with different NMDAR modulators. **A**, **B** Representative micrographs of neuronal dendrite taken < 100 µm from the soma of 1- and 5-day treatment, respectively. Green, synapsin; red, psd95; and blue, map2. **C**–**E** Quantification of synapse density (synapsin and psd95 colocalized puncta), presynaptic density (synapsin puncta), and postsynaptic density (psd95 puncta) from branches proximal (< 100 µm; **B**–**D**) and distal (> 100 µm; **F**–**H**) from the soma. Data are represented as means ± S.D. *n* = 30–39 (for close to the soma) and 8–20 (for far from the soma) randomly chosen neurons per treatment from three biological replicas. One-way ANOVA followed by Tukey’s post hoc. Scale bar = 2 μm

To find out if dendritic field expansion by NMDAR modulators is associated with changes in dendritic spine morphology and density of excitatory synaptic inputs, we treated cortical cultures as described above and stained neurons for dendritic spines and presynaptic-postsynaptic markers. Our attempts to compare different spine morphologies using phalloidin staining were unsuccessful due to ambiguities over morphological differences of spines captured with our settings and rapid bleaching of the fluorescence signal (Fig. 1G–I). Figure 4A and B shows typical examples of synapsin, psd95, and map2 triple-stained samples. Because remodelling effects of endogenous NMDAR modulators on dendrites depend on somatic proximity, we investigated if there is differential sensitivity of synaptic inputs in proximal (< 100 mm) vs. distal (> 100 mm) dendritic compartments (Fig. 4C–E vs. F–H). Analysis of psd95 and synapsin puncta density as well as synaptic contacts (co-localization of synapsin and psd95) in proximal dendritic segments showed an age-dependent increase of synapsin (two-way ANOVA; *F*_(1,1)_ = 30.699, *p* < 0.001) and psd95 (two-way ANOVA; *F*_(1,1)_ = 4.53, *p* = 0.034) puncta (Fig. 4D, E) as well as double-label contacts in all groups (two-way ANOVA; *F*_(1,1)_ = 15.961, *p* < 0.001) (Fig. 4C). Of note, the density of psd95 puncta was significantly reduced by PS-treated cultures (two-way ANOVA; *F*_(1,4)_ = 2.81, *p* = 0.026; Tukey’s post hoc, *p* = 0.040), as compared to naïve control. Comparison of the same immunofluorescence features of distal dendrites (> 100 mm) revealed no difference (Fig. 4F-H).

## Functional Impact of Dendritic Remodelling on the Activity of Cortical Neurons In Silico

**Fig. 5:**
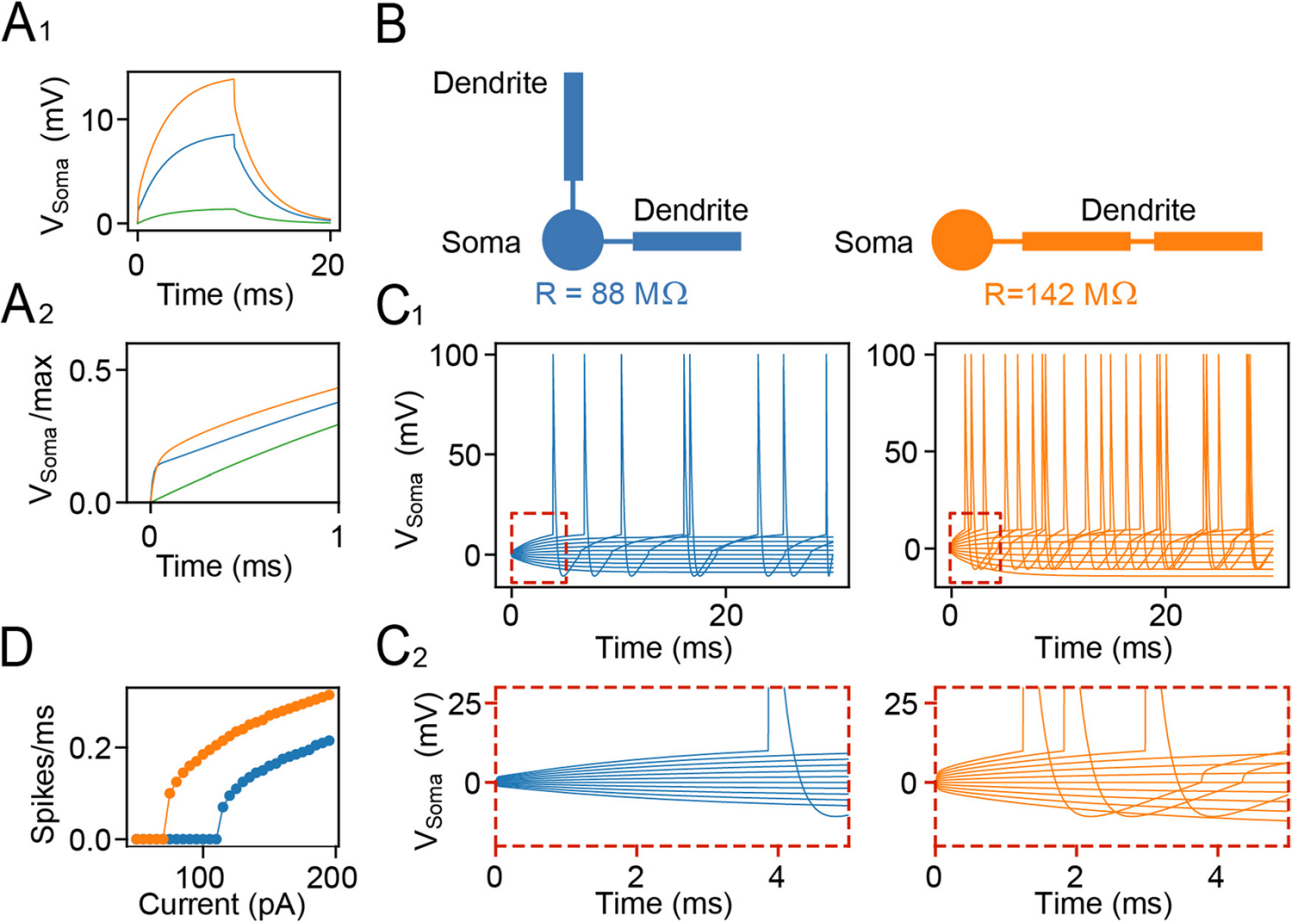
The altered morphology of cortical neurons changes their intrinsic excitability in a computational model. **A**_**1**_ Voltage responses to a 10-ms current pulse (with amplitude 100pA) for three model variants of a cell with the same surface and conductance density (green, single somatic compartment; orange, blue: morphologies shown in **B**). **A**_**2**_ Peak normalized responses from **A**_**1**_ in the first millisecond. **B** Morphologies consist of one somatic (circles) and two dendritic compartments (rectangles) that are either both linked to the soma (left: blue) or concatenated (right: orange), replicating the proximal or distal branching of dendrites. The somatic input resistance *R* is obtained from 100-pA current stimulation. **C**_**1**_ Voltage responses for varying input current amplitude in both model variants from **B** (colors). **C**_**2**_ Zoom into traces from **C**_**1**_. **D** F-I curve derived from simulations like in **C**_**1**_ with stimulus length 200 ms

The expansion of the dendritic tree without changes in the total length (surface area) of dendrites can have at least two effects on cortical neurons: (1) alter the geometry and distribution of synaptic inputs [27] and (2) affect the intrinsic excitability and regenerative activity [28]. Whereas the former cannot be realistically simulated in a neuronal culture model, alterations in intrinsic excitability and firing independently of the integration of the neurons into a neural circuit can be readily simulated, predicting the functional impact of described morphological changes. To this end, we constructed a computer model of a neuron with passive membrane properties in one somatic and two dendritic compartments and simulated the voltage response (see “Materials and Methods” section and Fig. 5A) for two morphological variants with an equal surface. In the first (control) variant, we connected the two dendritic elements directly to the soma, while in the second (experimental) variant, the two dendrites were concatenated (Fig. 5B) replicating an expanded dendrite. Upon direct somatic stimulation by incrementing rectangular currents, the voltage response of the neuron showed two-time constants, one reflecting the fast charging of the soma and the second, slower time constant, reflecting the charging of dendrites. Notably, the second component responded strongly to changes in dendritic configuration (Fig. 5A). As expected, the dendritic geometry strongly influenced the regenerative activity of neurons, with the concatenated model increasing the apparent somatic membrane resistance, impeding current flux into the dendrite. Together, the extended dendritic surface distally from the soma with stronger electrotonic somatic isolation (Fig. 5B, right) rendered the model neuron more responsive to somatic inputs leading to increased firing activity (Fig. 5C, D). The expansion of the dendritic field caused by endogenous modulators of NMDAR thus can have a direct impact on cellular excitability, with potential  knock-on effects of synaptic integration and cortical network dynamics.

## Discussion

Impairments of glutamatergic neurotransmission have been implicated in several neurological and psychiatric disorders, with NMDAR dysfunctions playing a key role. There is a major gap of knowledge on how molecular and functional changes in glutamatergic synaptic inputs translate into neurobehavioral phenotypes of mood disorders, addiction, as well as a range of developmental brain diseases such as autism and schizophrenia [13, 29–31]. Rising evidence indicates the potential significance of abnormal NMDAR-mediated synaptic activity in the pathobiology of these complex disorders, with schizophrenia among the most widely discussed [15]. Produced and released from neurons and glial cells, endogenous modulators of NMDAR emerge to have wide-ranging effects on neuronal processes and functions, with changes in their level in the cerebrospinal fluid and peripheral circulation reported in patients with schizophrenia [15].

We have investigated the impact of brief and prolonged exposure of rat cortical neurons to physiological concentrations of the most prevalent endogenous NMDAR modulators on excitatory synaptic inputs, evoked glutamate release, and complexity and morphology of dendritic tree in vitro. Our data show that NMDAR-mediated activity plays an important role in regulating the extent of dendritic projections and branching, with its reduction leading to the reduction of the dendritic field expansion, which could account for altered excitability and integrative properties of cortical neurons [32–34]. A decrease in dendritic tree expansion and altered pattern of  branching seems to be a specific response to examined NMDAR modulators, as we observed no changes in several other morphometric parameters. Importantly, in all tests, depolarization-induced glutamate release measured in the presence of DL-TBOA (inhibitor of glutamate clearance) [26] remained unchanged, suggesting that the overall number of glutamatergic connections remained unchanged. It is worth noting that the effects of dendritic arborization were most pronounced in cultures treated with spermidine and pregnenolone sulfate, with the directionality of dendritic morphology changes in all tests depending on the distance from the neuronal soma. Likewise, in all tests, enhancement of dendritic branching in distal compartments was associated with the depletion of more proximal dendrites. Intriguingly, in addition to shared effects on dendritic tree expansion, pregnenolone sulfate also caused a reduction in the density of the PSD-95–positive postsynaptic marker, implying also changes in synaptic integration processes.

The expansion in dendritic field area by NMDAR modulators is in general agreement with the well-documented role of this glutamatergic receptor in the regulation of structural and functional synaptic plasticity of cortical neurons, as shown by genetic or pharmacological studies [35–39]. To the best of our knowledge, the role of endogenous NMDAR modulators in shaping the geometry and the extent of dendritic projections has not been shown previously. Although the underlying processes of observed changes warrant further analysis, there is overwhelming evidence implying the key role of Ca^2+^ influx with activation of downstream signalling in regulating the complexity of the dendritic tree of neurons [33, 38, 40], with the involvement of spontaneous and evoked glutamate release and NMDAR-mediated effects [41, 42]. Of note, stimulation-induced NMDAR-dependent changes in glutamatergic connections appear to be limited to specific synaptic contacts and depend on Ca^2+^ calmodulin-dependent protein kinases II (CaMKII) [43–45], whereas remodelling of dendritic branching and dendritic tree complexity appear to rely on long-range volume transmission (paracrine) effects [38, 39]. Despite rapid clearance of released glutamate, it was shown to drive discrete intracellular Ca^2+^ waves in pyramidal neurons at distances in a range of tens of microns from the release site [38]. It is worth stressing that the effects of NMDAR activity on dendritic complexity might depend on multiple variables, with the NMDAR blockade known to restrict dendritic growth [46, 47] and promote dendritic branching [48]. The NMDAR-mediated effects on morphological plasticity, thus, can be confined to postsynaptic compartments and spines, mediated via changes in the activity of local structural proteins [44, 49], or may activate long-range molecular signalling mechanisms, through mitogen-activated protein kinase (MAPK) and transcription factors with gene expression, leading to global response, including activity-dependent hetero-synaptic remodelling of remote dendritic spines and dendritic branches [40, 41]. The structural changes observed in our experiments, thus, could result from diffuse effects of glutamate [38, 39] controlled by endogenous NMDAR modulators.

In the context of the current discussion, it is important to note that endogenous NMDAR modulators used in our experiments can interact also with several other receptors and ion channels also implicated in synaptic transmission and plasticity mechanisms. KYNA, for instance, can inhibit AMPA and α7 nicotinic receptor–mediated effects [50–52]. Likewise, pregnenolone sulfate is known to modulate GABA_A_ and TRPM1/M3 [53, 54], while polyamines can suppress inward rectifier K^+^ channels [55] and serve as a ligand for Ca^2+^-sensing receptors [56]. Although it is possible that the effects reported in this study also involve non-NMDAR mechanisms, the similarity of the phenotypes induced by all tested modulators entails shared receptors. Alterations in dendritic geometry and changes in branching by endogenous NMDAR modulators with unchanged synaptic density advocate independent regulation of these important facets of dendritic morphology and functions [40, 57, 58]. As shown by the results of our computer model, changes in dendritic geometry without alterations in synaptic inputs can alter passive properties of cortical neurons. As a result, neurons become more responsive to synaptic inputs targeting soma and proximal dendrites, altering their integrative properties and firing activity [33, 59]. The expansion of the dendritic tree with sparse proximal branching would also alter the somatodendritic coupling, mitigating the invasion of back-propagating action potentials, with effects on retrograde signalling and synaptic plasticity mechanisms [60–63]. In expanded dendritic branches, these changes can also alter the response triggered by local inputs and propagating bursts of somatic action potentials [57, 64], decoupling dendrites from the soma, and altering subthreshold integration [64, 65]. Described changes in dendritic geometry, thus, uncover previously unknown terrains for future experimental and computations studies, which might shed light on the mechanisms of dendritic retardation and cortical thinning in schizophrenia, providing clues for understanding the neurobiological mechanism behind the manifestation of this complex disorder.

## Data Availability

All data supporting described findings can be obtained from the corresponding author (Saak V. Ovsepian, University of Greenwich) upon reasonable request. The material cannot be deposited in the public domain before publication.
